# Hepatorenal protective role of encapsulated *Holothuria atra* in a mice model of *Plasmodium berghei* ANKA inoculation

**DOI:** 10.5455/javar.2026.m1010

**Published:** 2026-03-05

**Authors:** Prawesty Diah Utami, Herin Setianingsih, Dewi Ratih Tirto Sari

**Affiliations:** 1 Parasitology Department, Faculty of Medicine, Hang Tuah University, Surabaya, East Java 60244, Indonesia; 2 Anatomy and Histology Department, Faculty of Medicine, Hang Tuah University, Surabaya, East Java 60244, Indonesia; 3 Pharmacy Department, Faculty of Health Science, Ibrahimy University, Situbondo, East Java 68374, Indonesia

**Keywords:** Malaria, Encapsulation;, *Holothuria atra*, Hepatorenal, Histopathology

## Abstract

**Objectives:** Malaria continues to pose a substantial, widespread health challenge, necessitating innovative therapeutic approaches to combat drug resistance and alleviate organ damage associated with infection. This study investigated the hepatorenal protective effects of encapsulated *Holothuria atra* in an experimental malaria mouse model caused by *Plasmodium berghei* ANKA.

**Materials and Methods:** Random assignment of forty-eight BALB/c mice resulted in eight distinct groups for the study: an untreated control group (a negative control), a chloroquine-treated group (positive control), and six combination therapy groups receiving chloroquine alongside encapsulated *H. atra* with varying gum Arabic/soy protein isolate (GA/SPI) ratios and dosages. Renal and hepatic histopathology were assessed.

**Results:** The chloroquine-only group (G2) exhibited the highest renal damage scores. Combination therapy, particularly with the 4:1 GA/SPI ratio at 100 mg/kg (G8), significantly reduced renal and hepatic damage compared with G2, approaching negative-control levels in several parameters. Statistical analysis revealed significant differences in histopathological scores among the groups.

**Conclusions:** Encapsulation enhanced the therapeutic efficacy of *H. atra*, with the 4:1 ratio optimizing the delivery of antioxidant and anti-inflammatory compounds, thus mitigating drug-induced and malaria-related organ toxicities. Encapsulated *H. atra* shows promise as a valuable adjunct in antimalarial therapy.

## 1. Introduction

The persistent global health challenge posed by malaria, a severe and potentially lethal parasitic infection caused by protozoans of the genus *Plasmodium*, necessitates the urgent development of novel therapeutic strategies [1, 2]. Transmitted via infected *Anopheles* mosquitoes, the significant toll of malaria is exemplified by the 263 million cases and 597,000 deaths documented across the globe in 2023, with children under five bearing a disproportionate burden [3]. The increasing prevalence of drug resistance further compounds this crisis, demanding innovative approaches that circumvent existing therapeutic limitations [4, 5].

Malaria infection, traditionally known for its effects on the bloodstream and nervous system, often leads to significant hepatic and renal impairment, which aggravates disease severity [6, 7]. This organ dysfunction results from a complex interplay of factors, including parasite sequestration in the microvasculature, an excessive immune response, and impaired blood circulation [8, 9]. Histologically, the hepatic tissue exhibits sinusoidal congestion, characterized by blood accumulation in the hepatic microvessels, along with an increased presence of Kupffer cells containing hemozoin pigment, a product of parasite metabolism. Hepatocellular necrosis may also be present to varying degrees [10]. In the kidney, glomerular congestion, tubular injury ranging from mild swelling to necrosis, and the formation of proteinaceous casts obstructing the tubules are commonly observed [11, 12].

Immunohistochemical techniques further reveal parasitic antigens, inflammatory mediators, and markers of cellular stress within these tissues [13, 14]. Notably, the extent of these histopathological alterations correlates with clinical severity, highlighting their potential as biomarkers for disease monitoring and therapeutic evaluation.

The black sea cucumber, *Holothuria atra*, has emerged as a notable subject of investigation within marine-derived natural products due to its rich bioactive profile [15, 16]. A previous study reported that *H. atra* contains nine terpenoid compounds, including 17-hydroxyfuscocineroside B, 24-dehydroechinoside B, Calcigeroside B, Echinoside A, Echinoside B, Fuscocineroside C, Holothurin A1, Holothurin A3, and Holothurin B. Despite its unremarkable appearance, *H. atra* contains compounds with demonstrated antimicrobial, anti-inflammatory, and antioxidant activities—key factors in promoting health and disease prevention [16, 17]. Preliminary findings also indicate potential applications in oncology, tissue regeneration, and neuroprotection [18, 19]. A computational investigation revealed the capacity of nine triterpenoid glycoside compounds to inhibit *Plasmodium falciparum* Orotidine 5-Monophosphate Decarboxylase Protein (PfOMPDC), a target for antimalarial drug development due to its role in the parasite’s metabolism [20]. Other bioactive compounds, such as coumaric acid, pyrogallol, ascorbic acid, chlorogenic acid, catechin, and rutin, have also been reported to exhibit antimalarial activity by inhibiting Falcipain-2 [21]. Although these promising outcomes require further validation, ongoing research underscores the importance of *H. atra* as a source of novel therapeutic agents.

Overcoming challenges related to stability, bioavailability, and targeted delivery of *H. atra* bioactive is critical for realizing their full therapeutic potential. Direct administration of crude extracts often results in limited efficacy due to compound degradation in the gastrointestinal tract, poor absorption across biological membranes, and non-specific distribution throughout the body [22, 23]. Encapsulation improves stability by shielding the active compounds from diverse environmental challenges, encompassing radiation exposure, thermal instability, enzymatic metabolism, and pH fluctuations encountered during transit through the digestive system [24, 25]. As a consequence of this barrier, enhanced bioavailability of encapsulated substances results in greater systemic exposure and a corresponding improvement in therapeutic results [24, 26]. Moreover, encapsulation can be tailored to promote targeted selective delivery to designated tissues or cells, maximizing drug concentration at the intended site while reducing off-target interactions [7, 27]. Our previous study reported that encapsulated Holothurin A1 and Echinoside were more effective antimalarial and antiplasmodial activities [28, 29]. Furthermore, encapsulated triterpene glycosides from *H. atra* showed higher antioxidant, antibacterial, and antiplasmodial activities [30].

The gum Arabic and Soy Protein isolate (SPI) matrix exemplifies an optimal encapsulation platform due to the synergistic properties of these components [31, 32]. Gum Arabic, a complex polysaccharide, provides exceptional emulsification efficiency, facilitating the formation of stable micro- or nano-emulsions that enhance the solubility and dispersibility of hydrophobic bioactives [33, 34]. Simultaneously, SPI, a plant-derived protein with amphiphilic characteristics, imparts mechanical strength, controlled-release properties, and mucoadhesive potential to the resulting encapsulating material, further improving drug absorption and prolonging residence time at the target site [35, 36].

Given the growing interest in natural products as sources of novel antimalarial agents, the encapsulation of *H. atra* offers a strategic avenue to harness its inherent bioactive potential. This approach not only facilitates improved drug delivery but also aligns with emerging evidence suggesting that synergistic interactions between synthetic drugs and natural compounds may enhance antimalarial efficacy and reduce the risk of drug resistance.

## 2. Materials and Methods

### 2.1. Ethical approval

This research employs a rigorous experimental methodology, using a post-intervention assessment with a control group or a post-test-only control group, conducted in a laboratory setting at the Institute of Tropical Diseases, Airlangga University. The study has obtained ethical approval from the Faculty of Medicine, Hang Tuah University, Ethics Committee No. I/030/UHT.KEPK.03/VII/2025, affirming its adherence to ethical standards. The sample consists of male BALB/c mice, which are well established in experimental animal studies.

### 2.2. Sample size

Sample size determination follows a specific formula, (t – 1) × (n – 1) > 15, with an additional 30% correction factor to account for potential variability or attrition [37]. For calculation:


1 \[
\begin{array}{l}
(8 - 1) \times ({\mathrm{n}} - 1) > 15\\
= 7\; \times ({\mathrm{n}} - 1) > 15\\
= \;({\mathrm{n}} - 1) > 15/7\\
= ({\mathrm{n}} - 1) > 2.14\\
{\mathrm{Therefore}},\,{\mathrm{n}} > 3.14
\end{array}
\]

Given that the sample size must be an integer, the minimum number of samples per group is 4. Considering the 30% correction factor, this initial figure is multiplied by 1.3, resulting in approximately 5.2. Since the sample size must be a whole number and to ensure sufficient power, the number of replicates per group is rounded up to 6. Consequently, the total number of mice used in this study amounts to 48.

### 2.3. Subjects and experimental design

This study involved a total of 48 seven- to eight-week-old male BALB/c mice, with body weights ranging from 20 to 25 gm. Mice were randomly assigned to eight treatment groups, with six animals per group. All mice were subsequently inoculated with *Plasmodium berghei* ANKA/PbA. Once parasitemia was established, the mice were further allocated into several groups according to the specified treatment regimens. Random assignment of mice resulted in eight treatment groups, each consisting of six animals:

- Group 1 (G1): Negative Control—Received only the CMC-Na 5% solution.
- Group 2 (G2): Positive Control—Administered chloroquine as a single agent.
- Group 3 (G3): Combination Therapy—Administered chloroquine with *H. atra* encapsulated with gum Arabic and SPI (1:4) at 25 mg/kg body weight.
- Group 4 (G4): Combination Therapy—Administered chloroquine in conjunction with *H. atra* encapsulated with gum Arabic and SPI (1:4) with a 50 mg/kg body weight.
- Group 5 (G5): Combination Therapy—Administered chloroquine in conjunction with *H. atra* encapsulated with gum Arabic and SPI (1:4) with a 100 mg/kg body weight.
- Group 6 (G6): Combination Therapy—Administered chloroquine with *H. atra* encapsulated with gum Arabic and SPI (4:1) at 25 mg/kg body weight.
- Group 7 (G7): Combination Therapy—Administered chloroquine with *H. atra* encapsulated with gum Arabic and SPI (4:1) at a 50 mg/kg body weight.
- Group 8 (G8): Combination Therapy—Administered chloroquine in conjunction with *H. atra* encapsulated with gum Arabic and SPI (4:1) at 100 mg/kg body weight.

*Inclusion criteria:* (1) Male BALB/c mice aged 7–8 weeks; (2) Healthy, with no clinical signs of illness or abnormal body weight; (3) Negative baseline parasitemia prior to infection; (4) Body weight within ± 20% of the group mean.

*Exclusion criteria:* (1) Any signs of illness, stress, or abnormal behavior before the study; (2) > 10% weight loss during acclimatization; (3) No parasitemia detected after inoculation; (4) Death unrelated to infection or the treatment protocol.

### 2.4. Extraction and encapsulation of Holothuria atra

*Holothuria atra* was extracted by maceration in 70% ethanol (Merck, Germany) overnight. Then the extract was filtered and re-macerated three times to obtain a high-yield extract. The *H. atra* solution was filtered and evaporated using rotary evaporation at 70°C and 500 rpm. Ethanolic extract of *H. atra* 0.5 gm was mixed with 2% of soy isolated protein in water and 1% gum Arabic solution in water with formula KGS14 (gum Arabic: soy isolated protein = 1:4) and KGS41 (gum Arabic: soy isolated protein = 4:1). The extract and encapsulant solution were stirred at 600 rpm for 120 min. The encapsulated extract was freeze-dried at –55°C under 0.02 mbar for 96 h. The encapsulated powder was characterized by Fourier transform infrared spectroscopy (FTIR) and scanning electron microscopy (SEM).

The rationale for using different ratios of gum Arabic (GA) and soy protein isolate (SPI) was based on their distinct physicochemical properties, which contribute to the encapsulation matrix. GA, a polysaccharide, provides strong emulsification and stability, while SPI, a protein with amphiphilic characteristics, enhances mechanical strength and controlled release. Two ratios (1:4 and 4:1) were selected to compare a protein-dominant versus a polysaccharide-dominant encapsulation system, aiming to identify the optimal release profile and bioactive stability. Therefore, the rationale for using GA and SPI ratios (1:4 and 4:1) was to compare polysaccharide- versus protein-dominant matrices for encapsulation efficiency and controlled release [31, 32].

The dosage of *Holothuria atra* used in this current study (25, 50, and 100 mg/kg body weight) was determined based on our previous toxicity and efficacy studies of the same extract, which demonstrated safety and pharmacological activity within this range [20, 21, 30]. These doses were also selected to evaluate the dose-dependent protective effects on hepatic and renal tissues.

### 2.5. Procedure for inducing malaria infection and administering treatment

The process of inoculating *Plasmodium berghei* ANKA (PbA) into the experimental mice involved injecting 0.2 ml of donor erythrocyte blood, containing a concentration of 1 × 10^6^ parasites, via the intraperitoneal route. Prior to inoculation, the parasitized blood was prepared in a sterile solution at an optimal concentration to accurately deliver the intended number of parasites. All steps were performed under aseptic conditions to avoid contamination and ensure experimental integrity [38]. Encapsulated *H. atra* extract therapy was administered starting from day 2 (48 h) post-inoculation, at which point the average parasitemia ranged from 2% to 2.10%.

### 2.6. Parasitemia quantification

Peter’s four-day suppressive test procedure was followed for performing the *in vivo* antimalarial assay [39]. From day 1 to day 4, parasitemia levels were measured serially after inoculation and during the 48-h observation period. Every day, the tail vein punctures were used to obtain blood samples [40]. On a glass slide, a thin blood smear was prepared, fixed with methanol, and stained for 15 min with 10% Giemsa solution. After that, the stained preparations were viewed at 1000× magnification using a light microscope. The percentage of infected erythrocytes among at least 1,000 erythrocytes was used to determine parasitemia [41].

### 2.7. Murine termination and hepatorenal tissue extraction

The standardized murine euthanasia protocol involves administering a lethal intraperitoneal dose of ketamine hydrochloride, calculated based on body weight [42]. After confirming deep anesthesia and cessation of vital signs, the mouse is placed in dorsal recumbence for a midline abdominal incision to expose and excise the hepatic and renal tissue.

Renal and hepatic tissues are rinsed with sterile PBS, then flash-frozen for molecular analysis or fixed in 10% neutral buffered formalin for histology and stored in labeled containers [43]. All procedures are performed using appropriate PPE and documented thoroughly, including the ketamine dose, administration route, and observations. Carcass and waste disposal follow institutional biohazard guidelines and adhere strictly to IACUC regulations.

### 2.8. Hepatorenal examination protocol

This protocol outlines a standardized approach for assessing histopathological changes in renal and hepatic tissue through microscopic analysis of prepared tissue sections. The evaluation includes identifying and semi-quantitatively grading common pathological lesions within the tissue. Histological slide preparation: The first step is to preserve the tissue by soaking it in 10% neutral buffered formalin for at least 24 h. This helps maintain the tissue›s structure. Tissue dehydration was achieved using a sequential alcohol series, followed by xylene clearing, and the tissues were then embedded in paraffin. This process makes the tissue firm enough to slice thinly. The paraffin-embedded tissue was sectioned into thin slices approximately 4–5 µm thick using a microtome. These sections are stained, primarily with the H&E staining method. This histological staining procedure facilitates the differentiation of various tissue components and enables the identification of any morphological abnormalities. Finally, the stained sections are permanently mounted onto glass slides utilizing a specialized mounting medium and coverslipped. This process protects tissue samples and facilitates microscopic examination [44].

Histopathological observations of hepatic and renal tissues subjected to treatment were conducted at 5 different fields of view (LP) at 400× magnification. The observations were performed directly on the images and systematically analyzed using the Image Raster software to obtain average values. These examinations use a Nikon Eclipse Ei light microscope enhanced by Optilab SIGMA MTN020, which is connected to a computer.

### 2.9. Interpretation of renal histopathology

Renal histopathological assessment involves independently scoring specific features within each field: (1) Cast Formation: Presence of cellular casts in renal tubules; (2). Glomerulonephritis: Infiltration of mononuclear inflammatory cells such as lymphocytes, monocytes, and macrophages within the glomeruli. (3) Tubular Necrosis: Evidence of tubular cell death due to stress, shown by nuclear changes and cell detachment into tubules; (4) Congestion: Blood accumulation causing swelling of renal vessels, including glomeruli and capillaries; (5) Tubular Dilatation: Expansion of tubular lumens caused by injury and fluid buildup [45, 46].

Each histopathological parameter is semi-quantitatively rated based on the extent of lesion involvement within the field of view using the following scale: (1) Score 0: No detectable pathological changes; normal tissue appearance; (2) Score 1: Minimal alterations affecting less than 10% of the tissue in the field; (3) Score 2: Mild lesions present but not predominant, affecting up to 20% of the tissue; (4) Score 3: Moderate involvement, with 21% to 50% of the tissue showing changes, possibly impacting function; (5) Score 4: Severe lesions affecting 51% to 75% of the tissue area; (6) Score 5: Extensive pathology involving more than 75% of the field, likely with significant functional impairment [47].

### 2.10. Interpretation of hepatic histopathology

Interpretation of hepatic histopathology involves evaluating several key features. These include the amount of lipid stored within hepatocytes, the number of Kupffer cells (the hepatic resident macrophages) per high-power field (HPF), and the extent of inflammatory cell infiltration in the portal tracts. Additionally, the assessment considers the number of bile ducts in the portal areas, the degree of blood accumulation in hepatic sinusoids, and the presence of hemozoin pigment within Kupffer cells or hepatocytes [10, 48]. Each parameter is evaluated semi-quantitatively using a standardized scale to assess the severity of hepatic injury [47].

## 3. Results

Serial parasitemia level assessments were conducted from day 0 (48 h after inoculation) until day 4 (24 h after the last administration of encapsulated *H. atra*). Encapsulated *H. atra* extract was administered on day 0 and continued through day 3. The measurement results are presented in Table 1.

**Table 1.** Parasitemia level assessment results.

Groups	Parasitemia level									
	Day-0	SD	Day 1	SD	Day 2	SD	Day 3	SD	Day 4	SD
G1	2.10	0.27	18.74	2.51	26.63	1.91	39.86	1.48	54.30	2.74
G2	2.08	0.47	3.43	0.81	4.60	0.86	2.60	0.23	0.00	0.00
G3	2.06	0.63	10.23	0.36	7.46	1.17	6.24	0.42	5.04	0.49
G4	2.09	0.24	9.34	0.96	6.21	1.07	4.60	0.84	3.77	0.63
G5	2.07	0.32	8.04	0.61	5.87	0.35	4.47	0.46	3.00	0.41
G6	2.08	0.33	8.73	1.05	7.16	1.17	5.57	0.98	4.30	0.40
G7	2.05	0.60	9.34	0.96	5.37	0.73	4.27	0.47	3.45	0.42
G8	2.08	0.32	7.48	1.19	5.37	0.53	4.30	0.42	2.60	0.35

G1 = Negative control (infection without any treatment); G2 = Positive control (infection with chloroquine); G3–G5 = Combination of chloroquine and encapsulated *H. atra* with KGS ratio 1:4 at doses of 25, 50, and 100 mg/kg BW, respectively; G6–G8 = Combination of chloroquine and encapsulated *H. atra* with KGS ratio 4:1 at doses of 25, 50, and 100 mg/kg BW, respectively.

**Figure 1.**
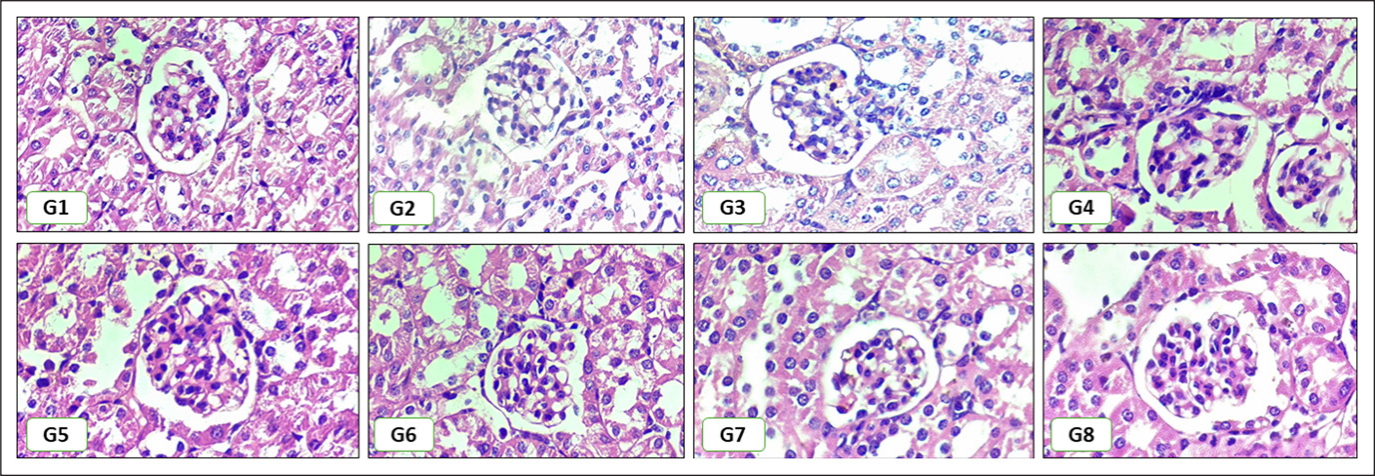
Renal Histopathological Change. Renal histopathology showed a varying degree of inflammation, congestion, necrosis, and tubular dilatation. G1: negative control; G2: positive control; G3-G5: chloroquine with encapsulated *H. atra* 25, 50, 100 mg/kg BW with GA/SPI 1:4; and G6–G8: chloroquine with encapsulation *H. atra* 25, 50, 100 mg/kg BW with GA/SPI 4:1. Magnification 400x. Scale bars are 50 µm.

The descriptive analysis (Table 2) indicates that the chloroquine-treated group (G2) had the highest renal damage scores. In contrast, the treatment group receiving the combination of chloroquine and encapsulated *H. atra* demonstrated a trend toward lower renal damage scores than G2, though still higher than those of Group G1, the negative control group, which received no treatment. The statistical analysis (Table 3) revealed a significant difference in renal damage scores among the groups; specifically, the control group (-)/G1 showed significantly lower scores compared to groups G2 through G6. Nevertheless, statistical analysis indicated that there was no significant difference between Group G1 (control) and Group G8, despite G8 having higher damage renal scores. Notably, the G8 group also showed a significantly lower damage score than G2, G4, G5, G6, and G7. The renal histopathological features are presented in Figure 1.

**Table 2.** Renal Histopathology features.

Groups	Mean	Median	S.D.	*p*-value
G1	1.430	1.500	0.294	0.0001
G2	3.070	3.300	0.700	
G3	1.830	1.900	0.197	
G4	2.500	2.500	0.452	
G5	2.120	2.100	0.329	
G6	2.430	2.300	0.638	
G7	2.000	2.000	0.551	
G8	1.630	1.500	0.320	

S.D. = Standard Deviation.

**Table 3.** Pairwise comparison results of the Mann-Whitney test among study groups in Renal Histopathology.

Study Groups (G)	1	2	3	4	5	6	7	8
1			**0.026**	**0.004**	**0.010**	**0.005**	**0.042**	0.411
2			**0.004**	0.107	**0.022**	0.170	**0.010**	**0.006**
3				**0.012**	0.094	**0.033**	0.667	0.164
4					0.164	0.627	0.094	**0.010**
5						0.371	0.359	**0.042**
6							0.166	**0.015**
7								0.188
8								

The Kruskal-Wallis test indicated a statistically significant difference in renal histopathological scores across all groups (*p* = 0.0001). The data analysis will be further performed using pairwise comparisons based on the Mann-Whitney U test to identify intergroup differences (Table 3).

**Figure 2.**
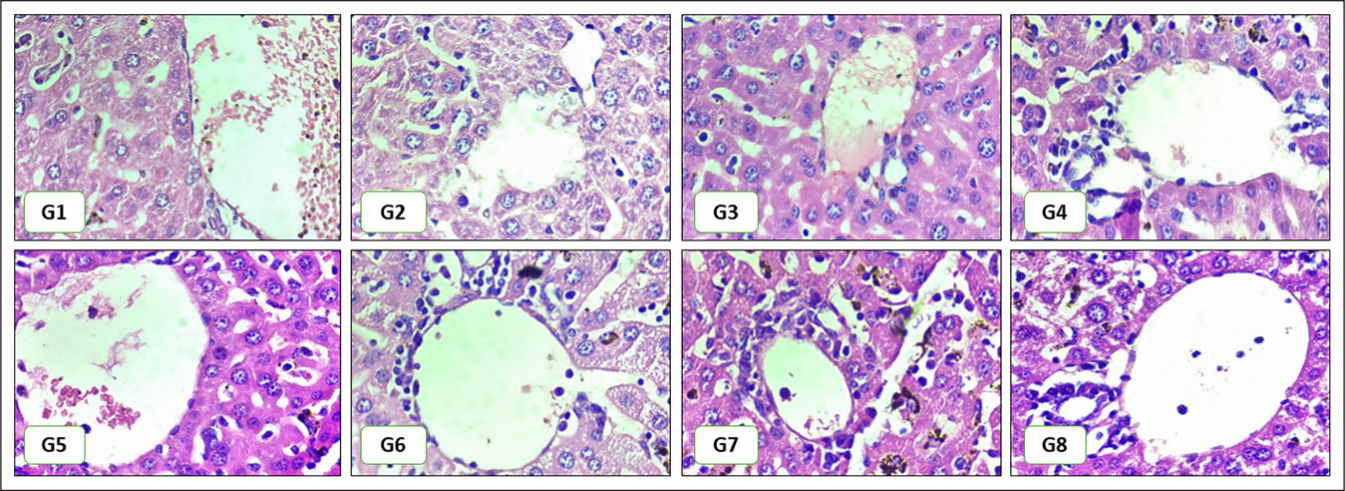
Hepatic Histopathological Change. Hepatic histopathology showed varying degrees of inflammation, sinusoidal congestion, Kupffer cell proliferation, and bile duct proliferation. G1: negative control; G2: positive control; G3-G5: chloroquine with encapsulated *H. atra* 25, 50, 100 mg/kg BW with GA/SPI 1:4; and G6–G8: chloroquine with encapsulation *H. atra* 25, 50, 100 mg/kg BW with GA/SPI 4:1. Magnification 400x. Scale bars are 50 µm.

The hepatic histopathological features presented in Figure 2 indicate that the lowest hepatic damage scores were observed in Group 8, followed by Group 1 (negative control). Conversely, the highest hepatic damage score was recorded in Group G3 (treated with chloroquine and encapsulated *H. atra*, GA/SPI 1:4, at 25 mg/kg BW), followed by Group 2 (treated with chloroquine). As shown in Table 4, the Kruskal-Wallis test demonstrated a significant difference in hepatic damage scores among the groups, with the negative control group (G1) exhibiting significantly lower cell damage scores compared to nearly all other groups, including the positive control and treated groups, except for Group G8. Although Group G8 showed higher hepatic damage scores than Group G1, this difference was not statistically significant. Additionally, the encapsulated *H. atra* with GA/SPI 4:1 at a dose of 100 mg/kg body weight demonstrated a significantly lower number of hepatic damages compared to Groups G2, G3, G4, G6, and G7.

**Table 4.** Liver Histopathology features.

Groups	Mean	Median	S.D.	*p*-value
G1	1.433	1.600	0.344	0.0001
G2	2.100	2.000	0.275	
G3	2.067	2.000	0.103	
G4	2.000	2.000	0.252	
G5	1.733	1.700	0.163	
G6	1.967	2.000	0.151	
G7	1.867	1.900	0.163	
G8	1.400	1.300	0.357	

S.D. = Standard Deviation.

Statistical analysis using the Kruskal-Wallis test revealed a significant difference in hepatic histopathological scores between the test groups (*p* = 0.0001), with the *p*-value falling below the alpha threshold of 0.05. Following this initial analysis, Mann-Whitney U tests will be performed for pairwise comparisons to identify specific differences between groups, as presented in Table 5.

**Table 5.** Pairwise comparison results of the Mann-Whitney test among study groups in Liver Histopathology.

Study Groups (G)	1	2	3	4	5	6	7	8
1		**0.003**	**0.003**	**0.010**	**0.101**	**0.005**	**0.016**	0.805
2			0.857	0.592	**0.016**	0.388	0.102	**0.011**
3				0.445	**0.007**	0.206	**0.031**	**0.008**
4					0.051	0.720	0.244	**0.016**
5						**0.037**	0.176	0.071
6							0.337	**0.018**
7								**0.033**
8								

## 4. Discussion

Statistical analysis of parasitemia levels on day 0, prior to treatment administration, revealed no significant differences among the treatment groups (*p* > 0.05). This lack of significant difference confirms that all groups had statistically equivalent and homogeneous baseline parasitemia levels prior to treatment. Consequently, baseline parasitemia conditions were uniform, ensuring unbiased interpretation of post-treatment observations.

Furthermore, the analysis demonstrated a dose-dependent decrease in parasitemia levels correlating with increasing doses of *H. atra*. This dose-dependent effect indicates that higher doses of *H. atra* extract exert a greater inhibitory effect on parasitemia, whereas lower doses correspond to reduced efficacy. Supporting this finding, Laksemi et al. [49] reported in an *in vivo* study that *Holothuria scabra* extract, an analog of *H. atra*, significantly inhibited the development of the *Plasmodium* parasite in animal models. Additionally, *in silico* studies have identified active compounds in *H. atra*, including chlorogenic acid and catechin, as inhibitors of Falcipain-2, a cysteine protease involved in the lifecycle of the malaria parasite, particularly in nutrient acquisition through erythrocyte protein degradation [49].

Although the combination of chloroquine and encapsulated *H. atra* extract did not achieve the same degree of parasitemia suppression as chloroquine monotherapy, histopathological examination demonstrated markedly reduced hepatic and renal injury in the combination group. This apparent dissociation between parasite burden and tissue damage suggests that *H. atra* exerts protective effects primarily through host-directed mechanisms rather than direct antiparasitic activity. Bioactive constituents of *H. atra*, including sulfated polysaccharides, triterpene glycosides, and phenolic compounds, possess potent antioxidants and anti-inflammatory properties that mitigate oxidative stress and inflammatory cascades commonly induced during malaria infection and chloroquine metabolism [50]. These compounds are known to enhance the activity of endogenous antioxidant enzymes (SOD, CAT, GPx), reduce lipid peroxidation (MDA), and down-regulate pro-inflammatory mediators such as *TNF-α, IL-1β*, and *IL-6* by suppressing NF-κB signaling [51, 52]. Consequently, hepatorenal cellular necrosis, tubular degeneration, and microvascular congestion are attenuated despite persistent parasitemia.

The descriptive analysis (Table 2) indicates that Group G2, treated with chloroquine, exhibited the highest renal damage scores. In contrast, the group receiving a combination of chloroquine and encapsulated *H. atra* showed a trend toward reduced kidney damage compared with G2, although it remained higher than in the negative control group (G1). The statistical analysis (Table 3) revealed significant differences, with the control group (G1) having markedly lower scores than G2-G6. However, no significant difference was observed between G8 and the other groups, despite its higher damage score. Notably, G8 demonstrated a significantly lower damage score compared to G2, G4, G5, G6, and G7. These findings align with previous studies reporting that marine-derived bioactive, particularly from sea cucumbers, exhibit antioxidant and anti-inflammatory properties that protect against drug-induced renal toxicity [53, 54]. Moreover, encapsulation strategies have been widely recognized as effective delivery systems for enhancing therapeutic outcomes and reducing organ toxicity [55].

The mechanism of renal damage due to malaria involves complex pathological processes triggered by *Plasmodium* sp. infection [12, 56]. Key pathways contributing to renal injury during malaria include: (1) hemolysis and erythrocyte rupture, along with the accumulation of hemozoin in the kidney; (2) disruptions in blood flow and hypoxia; (3) the activation of the immune system alongside inflammatory processes; and (4) toxic factors and oxidative stress [12]. Malaria parasites infect erythrocytes, leading to widespread hemolysis and the release of hemoglobin. Incomplete degradation of hemoglobin results in the formation of hemozoin, which can accumulate within renal tissues. This accumulation of hemozoin induces cellular damage and exacerbates oxidative stress in the kidneys [57, 58]. Additionally, infection causes vasoconstriction and impaired renal perfusion, leading to renal cell hypoxia and an increased risk of tubular necrosis and vascular injury [45, 46]. The infection also triggers an intense immune response characterized by the release of pro-inflammatory cytokines and other mediators. Consequently, this results in inflammatory cell infiltration, edema, and structural damage within the kidney, including glomerulonephritis [59]. Reactive oxygen species production is driven by oxidative stress induced by hemoglobin degradation and immune activation, leading to membrane damage, lipid peroxidation, and apoptosis of tubular and glomerular cells [56, 60].

Group G2, which was infected with malaria and treated with chloroquine, exhibited the highest and statistically significant renal damage scores among all groups, including the negative control (G1) and other treatment groups. All treatment groups receiving the combination of chloroquine and encapsulated *H. atra* also exhibited some degree of renal damage. This phenomenon may occur due to the complex interaction between the drugs’ therapeutic effects and the pathophysiological processes induced by the infection. These findings align with previous studies suggesting that infections with *Plasmodium falciparum* and *Plasmodium vivax* increase oxidative stress by elevating the production of reactive oxygen species (ROS) and reactive nitrogen species (RNS). This heightened oxidative environment triggers the induction of macrophages and dendritic cells, the secretion of inflammatory cytokines, and subsequent tissue damage resulting from excessive inflammation. Furthermore, antimalarial drugs such as artemisinin and quinoline derivatives exploit elevated ROS levels to eliminate parasites; however, this mechanism can also cause collateral damage to host tissues [8, 61].

Notably, the combination therapy of chloroquine with *H. atra* encapsulated in gum Arabic and soy protein isolate (SPI) at specific ratios demonstrated remarkable renoprotection. Treatment with the 4:1 ratio at 100 mg/kg BW (G8) resulted in histological scores (1.63) comparable to those of the negative control, indicating reduced renal tissue lesions. This suggests that the synergistic interaction between chloroquine and bioactive compounds in *H. atra*, enhanced through encapsulation technology, can mitigate renal injury. Encapsulation with gum Arabic and SPI with a 4:1 ratio likely improved the stability, bioavailability, and controlled release of the active constituents, thereby optimizing their therapeutic efficacy [31, 62]. These findings align with previous studies reporting that marine-derived bioactives, particularly from sea cucumbers, exhibit antioxidant and anti-inflammatory properties that protect against drug-induced renal toxicity [54]. Moreover, encapsulation strategies have been widely recognized as effective delivery systems for enhancing therapeutic outcomes and reducing organ toxicity [55, 63].

The histological features of the hepatic tissue revealed variable pathological profiles across the treatment groups. Compared to the negative control (G1), all experimental groups (G2–G7) exhibited elevated scores of fatty changes, portal tract inflammation, bile duct proliferation, and sinusoidal congestion, indicating hepatic injury. The increase in Kupffer cell count per high-power field (hpf) and hemozoin deposition in these groups further reflects heightened inflammatory and degenerative responses within the hepatic microenvironment. These alterations are consistent with hepatic pathology associated with drug-induced stress and malaria-related complications [10, 64]. Among the groups, the combination therapy of chloroquine with *H. atra* encapsulated in gum Arabic and soy protein isolate (SPI) at a 4:1 ratio and 100 mg/kg BW (G8) markedly attenuated hepatic injury. This treatment significantly reduced hepatic damage compared with other groups, yielding a histological profile closer to that of the negative control. These outcomes highlight the potential hepatoprotective effect of *H. atra* bioactive when delivered through encapsulation technology.

The reduction in hepatic alterations may be attributed to the antioxidant, anti-inflammatory, and immunomodulatory activities of bioactive compounds in *H. atra*. Previous studies demonstrated that sea cucumber-derived triterpene glycosides and phenolic compounds exhibit strong free radical scavenging and anti-inflammatory effects, thereby attenuating hepatic tissue damage [15, 65]. Furthermore, the 4:1 ratio indicates a higher proportion of gum Arabic, which serves as a stable encapsulation matrix that delays the release of the active compounds. This prolongs the duration of action of the active constituents of *H. atra* within the body, thereby allowing their antioxidant and hepatoprotective effects to function more effectively against oxidative stress and liver inflammation. Consequently, hepatic damage can be suppressed more efficiently [66].

The encapsulation ratio significantly influences its stability and solubility, with the 4:1 ratio providing superior protection against enzymatic and oxidative degradation, thereby maintaining active compound efficacy during treatment [62, 67]. In contrast, the 1:4 ratios, with higher SPI and lower gum Arabic content, offer less optimal protection and release, resulting in reduced effectiveness against fatty liver changes. These findings highlight the crucial role of carrier ratio optimization in formulation development to enhance stability, release, and therapeutic efficacy of *H. atra* in mitigating hepatic damage during malaria.

The observed improvement in hepatic tissue integrity across treatment groups demonstrates a dose-dependent effect of encapsulated *H. atra*. Specifically, higher doses of the encapsulated extract correlated with a more pronounced preventive effect against tissue damage in both the liver and the kidneys compared to lower doses [68]. This suggests that the therapeutic benefits of *H. atra* depend on the concentration of active compounds delivered to the target tissues. As the dosage increases, a greater quantity of bioactive molecules, such as antioxidants and anti-inflammatory agents, likely becomes available to counteract the pathophysiological mechanisms induced by malaria and/or the administered chloroquine. The enhanced efficacy observed at higher doses indicates that achieving a sufficient concentration of *H. atra*’s protective constituents is critical for effectively mitigating tissue damage in this experimental context.

These findings align with the concept that combination therapy using standard antimalarials and natural marine-derived agents can mitigate drug-induced hepatotoxicity and parasite-related hepatic pathology. Thus, encapsulated *H. atra* in combination with chloroquine presents a promising strategy for protecting hepatic function during antimalarial therapy.

In this study, the group infected only with *Plasmodium berghei* ANKA (G1) exhibited less hepatic and renal injury than the chloroquine-treated groups (G2–G7). This observation suggests that the histopathological damage observed in the chloroquine-exposed animals may not be solely attributable to infection but could also reflect the hepatotoxic and nephrotoxic potential of chloroquine itself [61]. Chloroquine has been shown to induce mild hepatotoxicity in mice by generating reactive oxygen species (ROS), which trigger lipid peroxidation and disrupt hepatocellular membrane integrity, leading to leakage of intracellular enzymes such as AST, ALT, and ALP into the circulation [69]. These oxidative mechanisms compromise hepatorenal function, and when combined with infection-induced stress, they can exacerbate tissue injury. Therefore, the more pronounced lesions in chloroquine-treated animals likely reflect the additive effect of drug-induced oxidative damage superimposed on malaria-associated inflammation.

In this study, the entire volume of intracardiac blood obtained from each experimental mice was used to measure inflammatory (TNF-α) and anti-inflammatory (Treg–FoxP3) markers. Because these assays require whole blood and peripheral immune cells rather than serum alone, the remaining volume was insufficient to perform additional biochemical evaluations. Consequently, standard renal function biomarkers (BUN and creatinine) and hepatic injury markers (AST/SGOT and ALT/SGPT) could not be assessed within the same animals.

The higher score of renal and liver histopathology in the treatment group (G3-G7) than the negative control groups might be induced by the immunomodulatory actions of both *H. atra* constituents and chloroquine, which can (i) facilitate parasite killing and (ii) simultaneously reshape liver-kidney histopathology during and immediately after clearance. Mechanistically, *H. atra* extracts contain sulfated polysaccharides (notably fucosylated chondroitin sulfate, FCS) and peptides/saponins that activate macrophages (pinocytosis/NO/*TNF-α/IL-1β/IL-6*) and engage pattern-recognition pathways, thereby enhancing phagocytosis of parasitized erythrocytes and debris—an effect that can synergize with chloroquine’s parasiticidal action to accelerate blood-stage clearance [70]. Such immune stimulation improves control of parasitemia but, in the short term, also increases leukocyte influx and mediator production within highly perfused organs (liver and kidney), yielding more conspicuous inflammatory and degenerative features on histology at a fixed sampling time [71].

In contrast, Group 8—administered the encapsulated *Holothuria atra* extract formulated with gum Arabic and soy protein isolate at a 4:1 ratio (100 mg/kg BW)—showed renal histopathology scores that were not significantly different from the negative control, indicating protection of renal architecture. Moreover, this group exhibited a marked reduction in parasitemia compared with the positive-control group receiving chloroquine alone. These findings suggest that microencapsulation not only preserved but also enhanced the bioactivity and bioavailability of *H. atra*’s bioactive compounds. The gum Arabic–soy protein matrix likely provided a synergistic encapsulation environment that stabilized antioxidant and anti-inflammatory molecules, enhanced gastrointestinal absorption, and facilitated sustained release at the site of action.

Importantly, the present findings cannot be interpreted as definitive evidence of complete normalization of hepatic and renal architecture. This caution is warranted, as several methodological limitations constrain the strength and certainty of this conclusion. First, the study did not include a non-infected baseline control group, which would have provided a true reference for normal histological appearance and allowed clearer differentiation between partial improvement and full restoration. Without this comparator, the extent to which the observed reductions in tissue damage approximate normal physiological morphology remains uncertain. Second, all assessments were performed at a single early time point—day 4 post-treatment—capturing only the acute phase of pathological change and therapeutic response. At this stage, inflammatory and oxidative processes may still be ongoing, and tissue repair mechanisms may not yet be fully evident, making it unlikely that complete histological normalization would be detectable [51]. Third, the absence of survival-time monitoring prevents evaluation of whether the observed improvements in organ histopathology represent sustained protection that translates into longer-term clinical benefit [72].

Taken together, the findings of this study suggest that encapsulated *H. atra* exhibits promising yet inconclusive hepatorenal-protective and antimalarial potential. The high-dose formulation, with an optimized ratio of gum Arabic to soy protein isolate, demonstrated meaningful reductions in parasitemia and substantial preservation of renal architecture, supporting its biological activity. However, lower-dose formulations and alternative encapsulation ratios produced more variable outcomes, suggesting that the therapeutic effect is highly dependent on dose, carrier composition, and release characteristics.

Finally, the study utilized only two encapsulation ratios and limited dose tiers for comparative evaluation. Broader optimization of encapsulation matrices, release kinetics, and pharmacokinetic profiling would be necessary to fully characterize the therapeutic potential of the formulated extract. Taken together, these limitations indicate that while the present findings provide promising preliminary evidence for the efficacy of encapsulated *H. atra*, further studies with extended follow-up, survival analysis, molecular validation, and broader formulation optimization are required to comprehensively define its therapeutic value.

## 5. Conclusions

The findings of this study demonstrate that the encapsulated *Holothuria atra* formulation at 100 mg/kg body weight with a gum Arabic–soy protein isolate ratio of 4:1 exerts the most pronounced hepatorenal protective profile among the regimens tested. This formulation was associated with the lowest hepatic histopathology scores and a concurrent reduction in renal injury scores, reflecting attenuation of hepatocellular degeneration, inflammatory changes, and renal tissue lesions. In addition, it achieved a reduction in parasitemia relative to chloroquine monotherapy and the other encapsulated doses. These results indicate that both dose and encapsulation composition are critical determinants of the therapeutic performance of *H. atra* and identify the 100 mg/kg, 4:1 gum Arabic–Soy protein isolate formulation as the most promising candidate for further preclinical evaluation.

## Data Availability

The data presented in this study are available from the corresponding author upon reasonable request.

## List of abbreviations

BW, body weight; CMC-Na, carboxymethyl cellulose Sodium; GA, gum Arabic; GI, gastrointestinal; H&E, hematoxylin and eosin; hpf, high power field; Hp, hepar (liver); IACUC, Institutional Animal Care and Use Committee; *IL-8*, interleukin-8; OMPDC/PfOMPDC, Orotidine 5′-monophosphate decarboxylase (*Plasmodium falciparum* OMPDC); PBS, Phosphate buffered saline; PbA, *Plasmodium berghei* ANKA; PPE, personal protective equipment; ROS, reactive oxygen species; RNS, reactive nitrogen species; SPI, Soy protein isolate; WHO, World Health Organization; mg/kg, milligrams per kilogram; min, minutes; h, hour.
